# LPS exacerbates TRPV4‐mediated itch through the intracellular TLR4‐PI3K signalling

**DOI:** 10.1111/jcmm.18509

**Published:** 2024-07-03

**Authors:** Yanping Hao, Liyan Wu, Yuhui Wang, Dongmei Shan, Yifei Liu, Jing Feng, Yi Chang, Ting Wang

**Affiliations:** ^1^ Shanghai Institute of Materia Medica, Chinese Academy of Sciences Shanghai China; ^2^ University of Chinese Academy of Sciences Beijing China; ^3^ Yangpu Hospital, School of Medicine Tongji University Shanghai China; ^4^ Department of Anesthesiology, Plastic Surgery Hospital Chinese Academy of Medical Sciences and Peking Union Medical College Beijing China; ^5^ Yunnan Key Laboratory of Gastrodia and Fungi Symbiotic Biology Zhaotong University Zhaotong Yunnan China; ^6^ Yunnan Engineering Research Center of Green Planting and Processing of Gastrodia Zhaotong University Zhaotong Yunnan China

**Keywords:** itch sensitization, LPS, PI3K, TLR4, TRPV4

## Abstract

Pruritus is often accompanied with bacterial infections, but the underlying mechanism is not fully understood. Although previous studies revealed that lipopolysaccharides (LPS) could directly activate TRPV4 channel and TRPV4 is involved in the generation of both acute itch and chronic itch, whether and how LPS affects TRPV4‐mediated itch sensation remains unclear. Here, we showed that LPS‐mediated TRPV4 sensitization exacerbated GSK101‐induced scratching behaviour in mice. Moreover, this effect was compromised in TLR4‐knockout mice, suggesting LPS acted through a TLR4‐dependent mechanism. Mechanistically, LPS enhanced GSK101‐evoked calcium influx in mouse ear skin cells and HEK293T cells transfected with TRPV4. Further, LPS sensitized TRPV4 channel through the intracellular TLR4‐PI3K‐AKT signalling. In summary, our study found a modulatory role of LPS in TRPV4 function and highlighted the TLR4‐TRPV4 interaction in itch signal amplification.

## 1 INTRODUCTION

Lipopolysaccharides (LPS), the major glycolipids of most gram‐negative bacterial outer membranes, is generally the most potent immunostimulant among these cell‐wall components.^1^ Toll‐like receptors (TLRs) sense LPS to activate the production of inflammatory mediators including cytokines, chemokines and histamine, ultimately leading to inflammation and pain.^2^, ^3^ Besides the canonical LPS receptor TLR4, recent studies revealed that transient receptor potential (TRP) channels may also serve as the molecular sensor for LPS.^4^, ^5^ For instance, bacteria directly activated nociceptors whereas key immune activation pathways were not necessary for hyperalgesia during acute infection.^6^ Recently, a role for TRPM4 has also been shown in LPS‐induced endothelial cell death.^7^ Intriguingly, S. aureus V8 activates mouse and human sensory neurons through proteinase‐activated receptor 1 (PAR1) to induce itch and scratch damage.^8^ One observation that LPS excites somatic and visceral nociceptor neurons via a mechanism dependent on TRPA1 activation and independent of TLR4 may readily explain acute pain and neurogenic inflammation induced by LPS,^3^ suggesting TRP channels are directly involved in the somatosensation in host‐pathogen interactions.

In addition to TRPA1, previous studies have shown that LPS also activates TRPV4 channel in airway epithelial cells and urothelial cells, mediating LPS‐induced increases in ciliary beat frequency and voiding frequency.^9^, ^10^ Nevertheless, our data argue against their conclusion that TRPV4 is the receptor for LPS,^11^ as we did not observe that LPS elicited a TRPV4‐mediated calcium influx in various cell lines, which was consistent with the fact that injection of LPS did not evoke any TRPV4‐dependent pain behaviour in mice.

It is strongly reminiscent of that LPS was not able to activate TRPV1 channels but sensitized TRPV1 via a TLR4‐mediated mechanism.^12^, ^13^ In general, the LPS‐TLR4 interaction might trigger intracellular signalling cascades in the nociceptors, leading to the sensitization of TRPV1, which likely underlies odontogenic infections, TNBS‐induced colitis, CIPN‐related hyperalgesia and histamine‐mediated pruritus.^13^, ^14^, ^15^, ^16^ Furthermore, it has been revealed that changes in extracellular matrix stiffness cooperating with LPS treatment triggered TRPV4 to mediate macrophage phagocytic function and lung infection.^17^, ^18^ Considering TRPV4 is also involved in the transduction of itch sensation, this provides a hint that TRPV4 may play a significant part in the process of LPS infection‐associated itch in mice, prompting us to investigate the role of the implication of LPS‐TRPV4 interaction in itch signal transduction.

Combining calcium imaging, behaviour test, quantitative polymerase chain reaction (qPCR), Western blot, as well as pharmacological and genetic strategies, we tested the hypothesis that LPS may enhance itch perception by the way of sensitizing TRPV4 channels. Surprisingly, we found that co‐application of LPS and GSK101 resulted in aggravated itch response in mice, which was abolished in TLR4‐deficient mice. Importantly, LPS‐mediated TRPV4 sensitization relied on TLR4‐induced intracellular PI3K activity. Altogether, these results demonstrate that LPS may modulate TRPV4‐dependent itch behaviour and TLR4‐PI3K‐AKT signalling is critically involved in the sensitization of TRPV4.

## 2 MATERIALS AND METHODS

### 2.1 Animals

All animal procedures were performed in accordance with the institutional ethical guidelines on animal care and approved by the Institutional Animal Care and Use Committee (IACUC) of Shanghai Institute of Materia Medica. Wild‐type C57BL/6J were obtained from the Jackson Laboratory (Bar Harbor, Me, USA). *Tlr4* ^*−/−*^ and *Trpv4* ^*−/−*^ mice were purchased from Cyagen and GemPharmatech, respectively. All mice were group‐housed in standard mouse housing cages at ambient temperature of 21–24°C with free access to food and water under a 12‐h light/dark cycle. Body weight‐ and sex‐matched mice were used in behaviour experiments at about 7–9 weeks of age and mice were randomly assigned to different experimental conditions.

### 2.2 Acute itch behaviour

Mice were shaved on the nape of the neck and acclimated for at least 3 days before assay. On the day of experiment, mice were habituated in the behavioural testing apparatus for 1 h by placing each of them individually in the recording chamber followed by intradermal injection of drugs in 50 μL sterile saline. Immediately after the injection, mice were videotaped for 30 min from a side angle without any person in the recording room. After the recording, the videotapes were played back and the number of scratching bouts toward the injection site was manually scored by an investigator blinded to the treatments or genotypes of animals.

### 2.3 Mechanical pain test

Mice were acclimated for 1.5–2 h in red plastic chambers on a metal mesh platform. LPS (5 μg/mL) was prepared in 0.9% sterile saline and delivered via intraplantar injection in a volume of 20 μL. Calibrated von Frey filaments of varying forces (0.02 g, 0.04 g, 0.07 g, 0.16 g, 0.4 g, 0.6 g, 1.0 g, 1.4 g and 2.0 g) were applied perpendicularly to the plantar surface of the hind paw for up to 2 s with sufficient force to bend the filament. Brisk withdrawal or paw flinching of the hind paw during or immediately after application was considered as a positive response. Mechanical hyperalgesia was evaluated before (baseline conditions) and 3 h after LPS injection (LPS treatment). The threshold force required to elicit withdrawal of the paw (50% mechanical threshold) was measured using the up‐down method.

### 2.4 HEK293T cell culture and transfection

HEK293T cells were grown as adherent monolayer maintained in Dulbecco modified Eagle medium (DMEM) (Gibco) complete medium (DMEM supplemented with 10% FBS (Gibco), 100 units/mL penicillin and 100 mg/mL streptomycin) in a humidified incubator at 37°C with 5% CO_2_. The cells were transiently transfected with mouse TRPV4 cDNAs using HighGene transfection reagent (ABclonal). Following transfection, the cells were maintained in DMEM for at least 24 h before use.

### 2.5 Single‐cell suspensions from mouse ear skin

Fresh mouse ears were cut and separated with forceps and digested in 0.25 mg/mL Liberase TL (Roche, Mannheim, Germany) in DMEM media for 90 min at 37°C. Samples were mashed through 70 μm cell strainers and washed with DMEM complete medium. Single‐cell suspensions were used for subsequent assays.

### 2.6 Live‐cell calcium imaging

4 μM Fura‐2 AM (Sigma‐Aldrich) was used to loading cells in culture medium for 60 min in the dark at 37°C. Before use, Fura‐2‐loaded cells were washed for at least 3 times with Hanks' Balanced Salt Solution (HBSS) at room temperature. Coverslips were moved to the standard external solution containing (in mM): 130 NaCl, 5 KCl, 2 CaCl_2_, 1 MgCl_2_, 30 glucose and 10 HEPES. Calcium imaging was performed on an Olympus BX51 microscope with Rolera Bolt camera (Q‐Imaging) and a CoolLED pE‐4000 (365/385) illumination system controlled via MetaFluor software (Molecular Devices). Fluorescence values were recorded at 340 nm and 380 nm excitation wavelengths. Fura‐2 ratios (F340/F380) reflecting changes in [Ca^2+^]_i_ upon stimulation were monitored and recorded. Values were obtained from 100 to 300 cells in time‐lapse images from each coverslip and cells were considered responsive if they demonstrated a change in fluorescence ratio >20% of baseline.

### 2.7 RNA extraction and quantitative RT–PCR

Total RNA was extracted with TRIzol reagent (Vazyme), according to manufacturer's instructions. qPCR reactions were carried out in a volume of 10 μL per reaction containing 5 μL SYBR Green master mix (2×) (Vazyme), 1.5 μL cDNA, 0.5 μL 10 nM primer mix and 3 μL water. Relative mRNA expression levels of different target gene compared to 18S were calculated using 2^−ΔΔCt^ methods. Primer sequences used for each gene were selected from Primer bank. Primer sequences (5′ to 3′) used were as follows:

**Table:** 

PI3K	CACTGGAGTCACCGGCTAC (forward)
	GACACTGTGAACACACTCTCG (reverse)
PKC	GTTTACCCGGCCAACGACT (forward)
	GGGCGATGAATTTGTGGTCTT (reverse)
18S	AACTTTCGATGGTAGTCGCCGT (forward)
	TCCTTGGATGTGGTAGCCGTTT (reverse)

### 2.8 Protein extraction and Western blot

Single‐cell suspensions prepared as described above, were treated with vehicle, LPS, GSK101, LPS combined with GSK101, LPS and GSK101 combined with Wortmannin or LPS and GSK101 combined with PI‐828 for 3 h. LPS, GSK101, Wortmannin and PI‐828 were used at the concentration of 10 μg/mL, 10 nM, 10 μM and 10 μM, respectively. After treatment, ice‐cold RIPA buffer (Meilunbio, China) with a cocktail of phosphatase inhibitor (Roche, Mannheim, Germany) and protease inhibitor (Sigma‐Aldrich) was used to lyse the cells. The concentration of the protein samples was determined by PierceTM Rapid Gold BCA Protein Assay Kit (Thermo Fisher Scientific, USA), and the protein samples were subjected to SDS‐PAGE and transferred to polyvinylidene difluoride (PVDF) membranes (Millipore, cat. no. IPVH00010), then the membranes were incubated with primary antibodies at 4°C overnight. The primary antibodies we used are as follows: anti‐β‐Actin (1:1000; cat. no. AC026, ABclonal), anti‐pPI3K (1:1000; cat. no. 4228 T, Cell Signaling Technology), anti‐PI3K (1:1000; cat. no. 4257 T, Cell Signaling Technology), anti‐pAKT (1:1000; cat. no. 4058 T, Cell Signaling Technology) and anti‐AKT (1:1000; cat. no. 4685S, Cell Signaling Technology). The next day, membranes were washed in TBS‐T and incubated with HRP‐conjugated secondary antibody (1:1000; cat. no. 7074S, Cell Signaling Technology) for 1.5 h. The GV 6000Pro II software was used to visualize the protein bands by chemiluminescence and the ImageJ software was used to measure the gray scale of the protein bands.

### 2.9 Statistics

All data were presented as mean ± standard error of the mean (SEM) for *n* independent observations unless otherwise indicated. Data from independent experiments were pooled when possible or represent at least two independent replicates. No data were excluded from statistical analyses, unless due to technical errors. Student's *t*‐test was used to analyse statistical significance between two groups and ANOVA tests with Tukey's post hoc analysis were used to test hypotheses about effects in multiple groups. All tests were carried out as 2‐tailed tests. Statistical analyses were performed using Prism 8 (GraphPad Software). Significance is labelled as: ****p < 0.0001, ***p < 0.001, **p < 0.01, *p < 0.05, n.s., not significant.

## 3 RESULTS

### 3.1 LPS enhanced GSK101‐elicited acute itch

**FIGURE 1:**
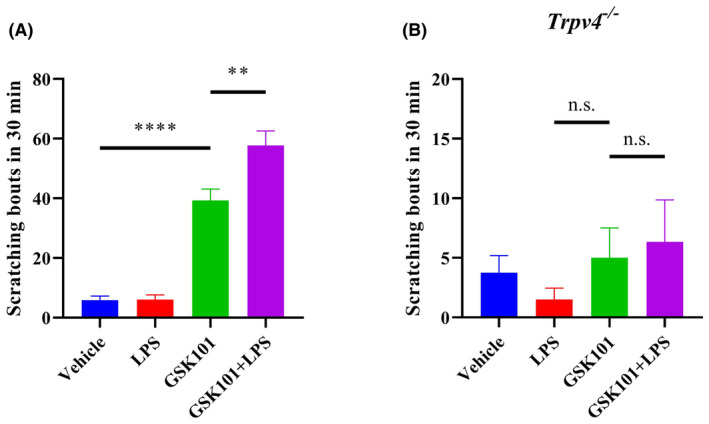
LPS aggravates GSK101‐induced itch sensation through TRPV4 channel. (A) Bar charts illustrated scratching responses produced by intradermal injections of vehicle (saline), LPS, GSK101 and GSK101 plus LPS (*n* = 6, **p < 0.01, ****p < 0.0001, one‐way ANOVA with Tukey's post hoc analysis). (B) TRPV4 KO mice were treated with vehicle, LPS, GSK101 or GSK101 combined with LPS and scratching bouts were counted for 30 min (*n* = 3–4, n.s., not significant, one‐way ANOVA with Tukey's post hoc analysis). LPS was used at the concentration of 10 μg/mL. 30 μM GSK101 was used to elicit acute itch behaviour.

First of all, we evaluated whether LPS was able to affect TRPV4 agonist GSK101‐induced acute scratching behaviour in mice. Interestingly, we found that LPS from *Salmonella enterica serotype abortus equi* could significantly increase GSK101‐induced scratching behaviour in wild type (*wt*) mice (Figure 1A). Moreover, neither vehicle control nor LPS alone could cause acute itching behaviour in *wt* and TRPV4 knockout mice (*Trpv4* ^*−/−*^) (Figure 1). As expected, GSK101 alone or in combination with LPS failed to evoke scratching events in TRPV4 KO mice, confirming that the itch behaviour caused by GSK101 is dependent on TRPV4 channel (Figure 1B). Collectively, our findings suggested that although LPS is not a potent pruritogen, it might sensitize TRPV4 channel to aggravate itch, driving us to explore the underlying mechanisms of LPS‐enhancing TRPV4‐mediated acute pruritus.

### 3.2 LPS sensitized TRPV4‐mediated calcium influx

**FIGURE 2:**
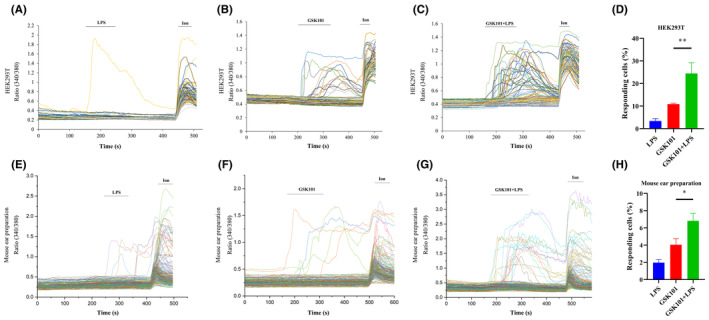
LPS enhances GSK101‐induced calcium influx in HEK293T cells and freshly isolated mouse ear skin single‐cell suspensions. (A–C) Representative time‐lapse traces illustrated that LPS could not evoke obvious [Ca^2+^]_i_ response in HEK293T cells transfected with TRPV4 construct (A, 32/952 cells), but increased GSK101‐elicited [Ca^2+^]_i_ responses of TRPV4 (B, 143/1321 cells and C, 373/1524 cells). (D) The bar chart described that percentage of HEK293T cells responded to LPS, GSK101 and LPS combined with GSK101 (**p < 0.01, one‐way ANOVA with Tukey's post hoc analysis). LPS was tested at the concentration of 10 μg/mL. 3 nM GSK101 was used to activate TRPV4 channels. (E–G) Representative time‐lapse traces showed that LPS did not evoke detectable [Ca^2+^]_i_ response in mouse ear single‐cell suspensions (E, 18/889 cells), but sensitized GSK101‐induced [Ca^2+^]_i_ responses of TRPV4 (F, 51/1253 cells and G, 105/1534 cells). (H) Summarized data represented percentages of mouse ear skin cells responding to LPS, GSK101 and LPS combined with GSK101 (*p < 0.05, one‐way ANOVA with Tukey's post hoc analysis). LPS was tested at the concentration of 10 μg/mL. 10 nM GSK101 was used to activate TRPV4 channels. Ionomycin (Ion) was used as positive control.

To investigate the mechanisms underlying the TRPV4‐potentiating effects of LPS at cellular level, we performed calcium imaging experiments. Strikingly, we found that although LPS did not evoke obvious intracellular Ca^2+^ ([Ca^2+^]_i_) response in HEK293T cells transfected with TRPV4, application of LPS sensitized TRPV4‐mediated [Ca^2+^]_i_ responses (Figure 2A–D). Similarly, LPS‐elicited [Ca^2+^]_i_ responses in GSK101‐treated cells were greater than GSK101‐evoked [Ca^2+^]_i_ responses in mouse ear single‐cell suspensions (Figure 2E–H). Together, these results indicated that LPS could sensitize TRPV4 channel in mouse ear single‐cell suspensions and heterologous expression HEK293T cells.

### 3.3 LPS potentiated TRPV4‐dependent itch via TLR4

**FIGURE 3:**
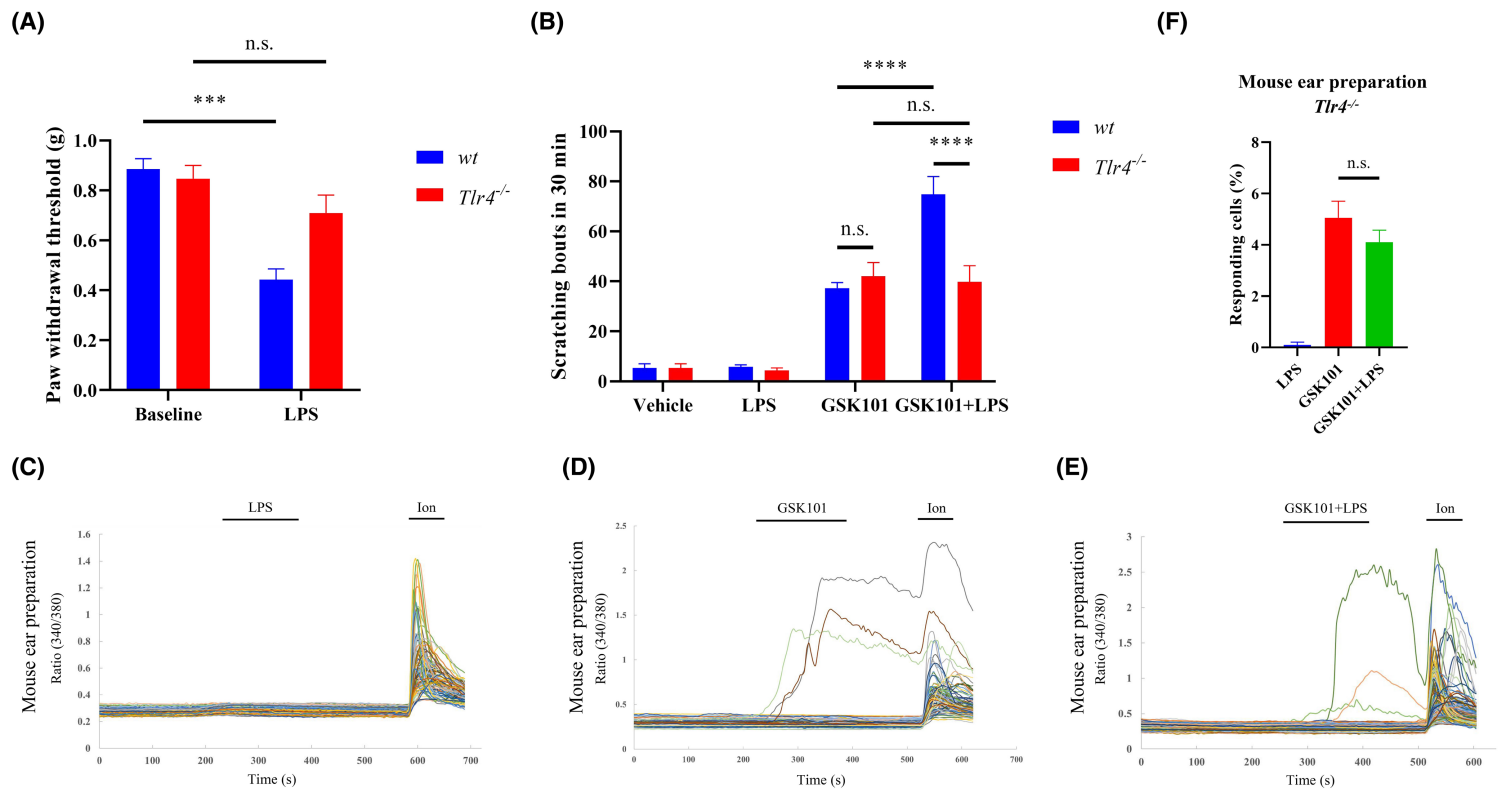
*Tlr4* ^*−/−*^ mice display markedly reduced LPS‐aggravated GSK101‐induced itch behaviour and [Ca^2+^]_i_ responses versus *wt* mice. (A) LPS‐induced mechanical hypersensitivity has no significant difference between *Tlr4* ^*−/−*^ and *wt* mice (*n* = 4, ***p < 0.001, n.s., not significant, two‐way ANOVA with Tukey's post hoc analysis). Intraplantar injection of LPS with 100 ng/20 μL. (B) LPS plus GSK101‐induced numbers of scratching bouts were comparably decreased in TLR4‐deficient mice. (*n* = 5–7, ****p < .0001, n.s., not significant, two‐way ANOVA with Tukey's post hoc analysis). (C–E) Representative time‐lapse traces illustrated that LPS could not evoke obvious [Ca^2+^]_i_ response in TLR4 deficient mouse ear single‐cell suspensions (C, 5/620 cells), and the enhancement effect of LPS on GSK101‐induced [Ca^2+^]_i_ responses was eliminated in *Tlr4* ^*−/−*^ mice (D, 34/692 cells and E, 28/687 cells). The concentration of LPS was 10 μg/mL and 10 nM GSK101 was applied. (F) Proportion of mouse ear skin cells of *Tlr4* ^*−/−*^ mice responded to LPS, GSK101 and LPS combined with GSK101 (n.s., not significant, one‐way ANOVA with Tukey's post hoc analysis).

TLR4 has been reported previously to mediated LPS‐elicited paw mechanical hyperalgesia in mice.^19^, ^20^ In addition, previous studies have demonstrated that TLR4 expression potentiates histamine‐mediated itch by regulating TRPV1 channel activity.^16^ We then tested if TLR4 is involved in the itch signal transmission triggered by the GSK101. Here we generated the TLR4 KO (*Tlr4* ^*−/−*^) mice and found that LPS injection evoked a robust mechanical pain in *wt* mice, while the paw withdrawal threshold was significantly increased in *Tlr4* ^*−/−*^ mice, suggesting an acceptable TLR4 KO efficiency (Figure 3A). To our surprise, increased scratching bouts caused by co‐application of LPS and GSK101 were severely reduced in *Tlr4* ^*−/−*^ mice (Figure 3B). What is more, the enhancement of TRPV4‐mediated calcium influx when applying LPS was eliminated in *Tlr4* ^*−/−*^ mouse ear skin single‐cell suspensions (Figure 3C–F). To sum up, LPS enhanced GSK101‐induced scratch responses in a TLR4‐dependent manner.

### 3.4 PI3K signalling is involved in LPS‐mediated TRPV4 sensitization

**FIGURE 4:**
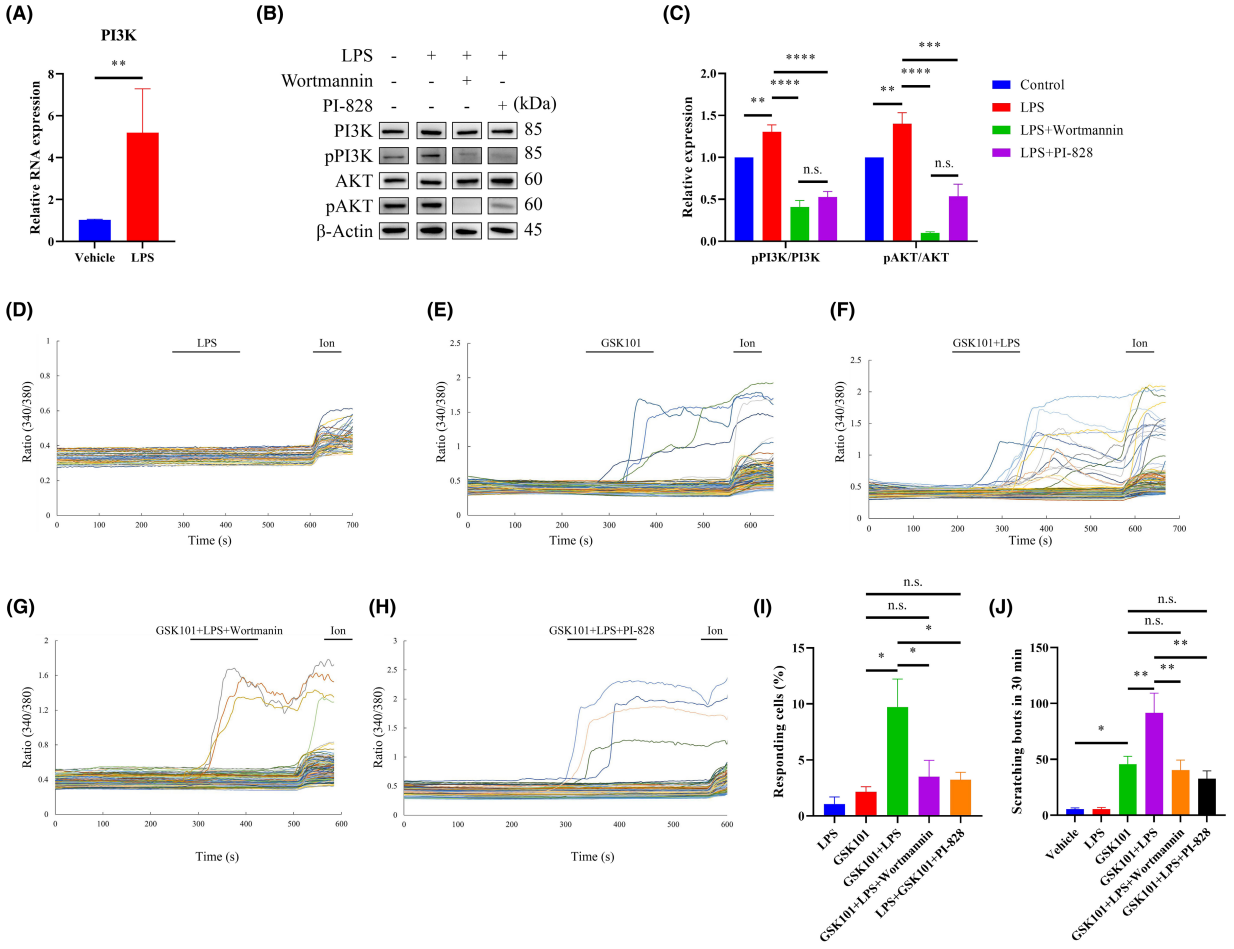
The effect of LPS on TRPV4‐mediated calcium influx and itch behaviour requires PI3K activity. (A) After pretreatment of mouse ear single‐cell suspensions with LPS for 3 h, the mRNA expression level of PI3K significantly increased (*n* = 3, **p < 0.01, *t*‐test). (B) Representative western blot experiments suggested that LPS up‐regulated the phosphorylation level of PI3K/AKT protein. β‐Actin expression was used as an internal control. (C) Quantitative analysis of grey density relative to β‐Actin of pPI3K/PI3K and pAKT/AKT (*n* = 3–6, n.s., not significant, **p < 0.01, ***p < 0.001, ****p < 0.0001, one‐way ANOVA with Tukey's post hoc analysis), and the expression of proteins were normalized to the control group. (D–H) Representative time‐lapse traces illustrated that LPS did not induce visible [Ca^2+^]_i_ response in mouse ear single‐cell suspensions (D, 6/637 cells), but sensitized GSK101‐induced [Ca^2+^]_i_ responses of TRPV4 (E, 14/705 cells and F, 76/774 cells), which could be abolished by wortmannin (G, 18/531 cells), a selective PI3K inhibitor, or PI‐828 (H, 21/661), a non‐specific PI3K inhibitor. (I) Statistical data of *wt* mouse ear skin cells responding to LPS, GSK101, LPS plus GSK101, LPS and GSK101 combined with wortmannin or PI‐828 (*p < 0.05, n.s., not significant, one‐way ANOVA with Tukey's post hoc analysis). LPS, GSK101, wortmannin, and PI‐828 were used at the concentration of 10 μg/mL, 10 nM, 10 μM and 10 μM, respectively. (J) LPS itself could not cause acute scratching behaviour in mice, but significantly increased GSK101‐induced number of scratching bouts and the enhancement was eliminated in the presence of PI3K inhibitors, wortmannin or PI‐828 (*n* = 4–8, *p < 0.05, **p < 0.01, n.s., not significant, one‐way ANOVA with Tukey's post hoc analysis). LPS was used at the concentration of 10 μg/mL, and 30 μM GSK101 was used to evoke acute itching behaviour, while the concentration of wortmannin and PI‐828 were used at 100 μM and 300 μM.

Prior studies demonstrated that TGF‐β induced the recruitment of TRPV4‐PI3K complexes to the plasma membrane and increased the function of TRPV4 channel.^21^ We supposed that LPS may trigger intracellular signalling cascades leading to the sensitization of TRPV4. Intriguingly, the expression of PI3K mRNA was significantly increased in mouse ear single‐cell suspensions after LPS treatment (Figure 4A). Additionally, we investigated the phosphorylation level of PI3K and its downstream effector AKT to confirm the involvement of PI3K signalling in the process of LPS‐evoked GSK101‐mediated TRPV4 sensitization. Consistent with the upregulated expression of PI3K mRNA, the phosphorylation level of PI3K/AKT was significantly elevated after treated with LPS (Figure 4B,C), which had no obvious difference compared to the control group in TLR4 deficient mouse ear skin cells (Figure S1). Moreover, the LPS‐induced enhanced expression of PI3K/AKT was markedly decreased in the presence of PI3K inhibitors, a selective inhibitor, wortmannin or a non‐specific inhibitor, PI‐828 (Figure 4B,C). It is noteworthy that the expression of pAKT was totally suppressed when the cells were treated with wortmannin (Figure 4B,C), which further confirmed that wortmannin is a selective inhibitor of PI3K with better selectivity than PI828. What is more, LPS did not evoke obvious [Ca^2+^]_i_ response, but sensitized GSK101‐induced [Ca^2+^]_i_ responses of TRPV4, which could be abolished by perfused with wortmannin or PI‐828 (Figure 4D–I), while the PI3K inhibitors themselves were not able to cause changes in intracellular calcium signalling acute scratching behaviour (Figures S2 and S3). Besides, pretreatment with intraperitoneal injection of wortmannin 30 min before applying GSK101/LPS mixture reversed the LPS‐induced itch sensitization (Figure 4J). Taken together, our data demonstrated that intracellular PI3K/AKT signalling contributed to the enhanced itch sensation of LPS in GSK101‐induced itch sensitization.

## 4 DISCUSSION

In this study, we showed that LPS from *Salmonella enterica serotype abortus equi* failed to evoke TRPV4‐dependent [Ca^2+^]_i_ response in freshly isolated mouse ear skin single‐cell suspensions or HEK293T cells transiently transfected with TRPV4. Nevertheless, LPS could sensitize GSK101‐induced [Ca^2+^]_i_ response and enhance scratching bouts in mice when co‐applied with GSK101. Our data revealed that TLR4, as the receptor for LPS, enhanced GSK101‐induced itch signal transduction by synergizing with PI3K/AKT pathway and potentiating TRPV4 activity to heighten itch sensation. To sum up, the results uncover that the LPS‐TLR4 signalling pathway mediates itch hypersensitivity in mice by regulating the activity of intracellular PI3K and affecting the function of TRPV4 channel, indicating that TLR4‐TRPV4 signalling could be a novel target for the treatment of infection‐related pruritus.

There is an alternative mechanism that the LPS‐activated TLR4 signalling pathway cooperates with TRPV4 to amplify itch signals. PLCγ2‐IP_3_‐Ca^2+^ signalling cascade is required for TLR4 endocytosis following LPS stimulation.^22^ The kinase PI3K mediates the subcellular compartmentalization of TLR4 signalling and protects from endotoxic shock.^23^ Significantly, TGF‐β stimulates PI3K to recruit TRPV4‐PI3K complexes to the plasma membrane, thereby increasing myofibroblast transdifferentiation.^21^ Consistent with our results, the LPS‐induced enhanced phosphorylation level of PI3K/AKT was markedly reversed when applying PI3K inhibitors or in TLR4 deficient mouse ear single cell suspensions (Figure 4B,C and Figure S1). Furthermore, PI3K inhibition abolished the effect of LPS in increasing TRPV4‐mediated scratching response and calcium influx (Figure 4D–J). We suspect that TRPV4 is recruited to the plasma membrane in response to PI3K activation to enhance itch sensation in the case of LPS infection. Apart from membrane trafficking, protein kinase C (PKC) pathway is also implicated in the sensitization of TRPV4. Phosphorylation of TRPV4 by PKC appears to be the predominant mechanism for channel activation.^24^, ^25^, ^26^ To determine whether LPS affects intracellular PKC expression, we detected PKC mRNA expression after LPS treatment and found no significant change in the PKC expression level (Figure S4), suggesting that LPS has an impact on the trafficking of TRPV4, rather than channel activation. In the further study, we will test the expression of TRPV4 channels in the cytoplasm and cell membrane after LPS treatment to ascertain our hypothesis.

TRPV4 is a ubiquitous mechanosensitive cation channel. In addition to being affected by intracellular signalling molecule, TRPV4 may be directly activated by sensing mechanical force changes caused by LPS. Previously reported that enhanced LPS‐induced inflammation was via stiffness‐dependent stimulation and LPS could stimulate changes in cellular mechanical properties in terms of elasticity and viscoelasticity.^27^, ^28^ It has been shown that LPS could directly induce cell swelling response in a TLR4‐dependent manner.^29^ Of note, TRPV4 regulates LPS‐stimulated macrophage phagocytosis in a matrix stiffness‐dependent manner.^17^ Likely, the mechanosensitive TRPV4 channel is activated by crystal‐induced cell volume change in LPS‐primed bone marrow derived macrophages.^30^ These results provide a clue that TRPV4 could be activated by LPS‐induced cell deformation. What is more, previous studies revealed that Piezo1 acts upstream of TRPV4 to induce pathological changes in endothelial cells and pancreatic acinar cells due to mechanical stimulation, and TLR4 signalling via Piezo1 plays a crucial role in the macrophage‐mediated pathogen ingestion and killing.^31^, ^32^, ^33^ Thus, TRPV4 might sense mechanical cues to transmit molecular signals involved in exacerbating GSK101‐induced itch sensation in the presence of LPS.

It is currently unclear which types of TRPV4‐expressing cells contribute to LPS‐induced augmented itch. Previous study showed that GSK101 elicits scratching‐behaviour dependent on TRPV4 expression in keratinocytes.^34^ Besides, it has been reported that Ca^2+^ is a second messenger in LPS‐mediated activation of the macrophage and TRPV4 regulates LPS‐stimulated macrophage phagocytosis.^17^, ^35^ Moreover, TRPV4‐expressing macrophages and keratinocytes contribute differentially to squaric acid dibutylester (SADBE)‐induced allergic and dry skin‐associated non‐allergic chronic itch.^36^ Additionally, mast cell degranulation releases a broad array of powerful mediators, such as histamine, proteases, cytokines and growth factors that can directly stimulate corresponding receptors on itch‐mediating sensory nerves to participate in the development of pruritus.^37^, ^38^ Previous research reported that TRPV4 plays a key role in cathelicidin‐driven mast cell activation in rosacea inflammation.^39^ Additionally, TRPV4 expressed in mouse and human skin is involved in the mechanism of pruritus in rosacea.^40^ In order to determine the mechanism of LPS‐induced acute itch exacerbations in TRPV4‐expressing cells, conditional knockout mice are particularly necessary in the future researches.

In addition to participating in acute itching, bacterial infections also contribute to the development of chronic pruritus. It was reported that the bacterial pathogen *Staphylococcus aureus* colonization has been found in 90% of the lesional skin of patients with atopic dermatitis.^41^ The potential mechanism of itch during bacterial infection is that *Staphylococcus aureus* releases deltatoxin and induces mast cell‐mediated pruriceptor activation in atopic dermatitis.^42^ Besides, LPS‐induced scratching responses of skin wound in mice model were much more serious than the control groups.^43^ TLR4, which recognizes LPS, plays a key role in itching.^16^, ^44^ Previous studies have reported that *Tlr4* ^*−/−*^ mice show reduced scratching in histamine‐mediated acute itch and in chronic itch models of dry‐skin pruritus, contact dermatitis and allergic contact dermatitis.^16^, ^44^ However, the contribution of LPS to pruritus remains controversial. Another study showed that topical application of LPS may alleviate allergic contact dermatitis.^45^ Moreover, the administration of LPS attenuated symptoms of atopic dermatitis in mice and patients.^46^, ^47^ The underlying mechanism by which LPS affects acute or chronic itch needs further experimental verification.

In summary, we found that LPS could not directly activate TRPV4, but enhanced the GSK101‐evoked acute itch by sensitizing TRPV4, and this sensitization relied on LPS‐TLR4‐PI3K‐AKT signalling pathway. This study expands our knowledge of the function of TRPV4‐expressing skin resident cells in the mechanisms of itchiness induced by LPS, sheds light on the clinical challenge of managing pruritus associated with bacterial infections, and suggests that targeting TRPV4 may provide a novel strategy for treating enhanced itch sensation in the course of inflammation.

## AUTHOR CONTRIBUTIONS

**Yanping Hao:** Investigation (lead); methodology (lead); writing – original draft (lead). **Liyan Wu:** Investigation (lead); methodology (lead); writing – original draft (lead). **Yuhui Wang:** Software (equal). **Dongmei Shan:** Visualization (equal). **Yifei Liu:** Supervision (equal). **Jing Feng:** Writing – review and editing (supporting). **Yi Chang:** Writing – review and editing (supporting). **Ting Wang:** Supervision (lead); writing – review and editing (lead).

## FUNDING INFORMATION

This work was supported by grants from the Research Project of Yunnan Key Laboratory of Gastrodia and Fungi Symbiotic Biology (grant no. TMKF2024B01), National Natural Science Foundation of China (grant nos. 82171214, 32241003), Natural Science Foundation of Shanghai (grant no. 23ZR1474500), Lingang Laboratory (grant no. LG‐QS‐202203‐07) and State Key Laboratory of Chemical Biology, Shanghai Institute of Materia Medica, Chinese Academy of Sciences, Shanghai, 201203, China.

## CONFLICT OF INTEREST STATEMENT

All authors have reviewed this manuscript and indicated that there are no conflicts of interest regarding the content of this manuscript.

## Supporting information


Figures S1–S4.

## Data Availability

All data contained in this document can be obtained from corresponding authors upon request.
