# Supplementary material for: LPS exacerbates TRPV4‐mediated itch through the intracellular TLR4‐PI3K signalling
Source: J Cell Mol Med. 2024 Jul 3;28(13):e18509. doi: 10.1111/jcmm.18509 (PMC11220342; doi:10.1111/jcmm.18509)
Supplement: Supplementary file 1 — Figures S1–S4. [file JCMM-28-e18509-s001.zip › Captions.docx]

SUPPLEMENTARY FIGURE 1 LPS treatment does not affect PI3K/AKT expression in Tlr4-/- mice. (A) When TLR4 deficient mouse ear cell suspensions were treated with or without 10 μg/mL LPS, the phosphorylation level of PI3K/AKT proteins had no significant difference. (B) Quantitative analysis of gray density relative to β-Actin of pPI3K/PI3K and pAKT/AKT (n=3, n.s., not significant, t-test).

SUPPLEMENTARY FIGURE 2 PI3K inhibitors wortmannin and PI-828 are independent of LPS-enhanced GSK101-mediated TRPV4 sensitization. (A-C) Representative time-lapse traces described that the effect of PI3K inhibitor wortmannin alone (A, 6/624) or combined with LPS (B, 5/619) or GSK101 (C, 43/683) on calcium influx response in mouse ear single-cell suspensions. (D) Percentage of mouse ear cells responded to wortmannin or wortmannin applying with LPS or GSK101. (E-G) Representative time-lapse traces suggested that the function of LPS (F, 9/637) or GSK101 (G, 35/694) was not influenced by PI-828 (E, 11/671). (H) Proportion of responding cells of PI-828 or PI-828 plus LPS or GSK101.

SUPPLEMENTARY FIGURE 3 PI3K inhibitors wortmannin and PI-828 do not affect the number of GSK101-induced scratching bouts. Wortmannin and PI-828 could not evoke acute scratching behavior and had no impact on GSK101-elicited TRPV4-mediated itch behavior (n=3).

SUPPLEMENTARY FIGURE 4 LPS treatment does not affect PKC mRNA expression. PKC mRNA expression in the freshly isolated mouse ear skin single-cell suspensions treated with vehicle or LPS (n=3, n.s., not significant, t-test).
