# Supplementary figures and images for: LPS exacerbates TRPV4‐mediated itch through the intracellular TLR4‐PI3K signalling
Source: J Cell Mol Med. 2024 Jul 3;28(13):e18509. doi: 10.1111/jcmm.18509 (PMC11220342; doi:10.1111/jcmm.18509)

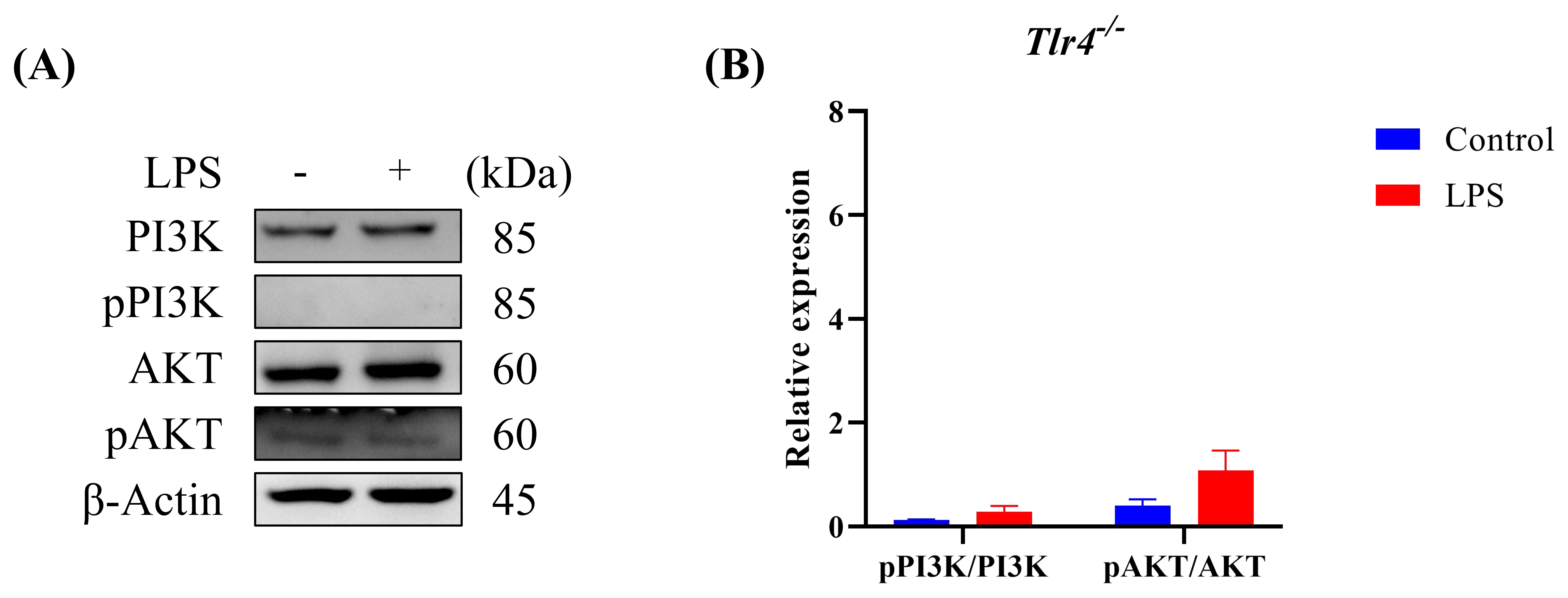

Supplement: Supplementary file 1 — Figures S1–S4. [file JCMM-28-e18509-s001.zip › jcmm18509-sup-0001-FigureS1 .jpg]

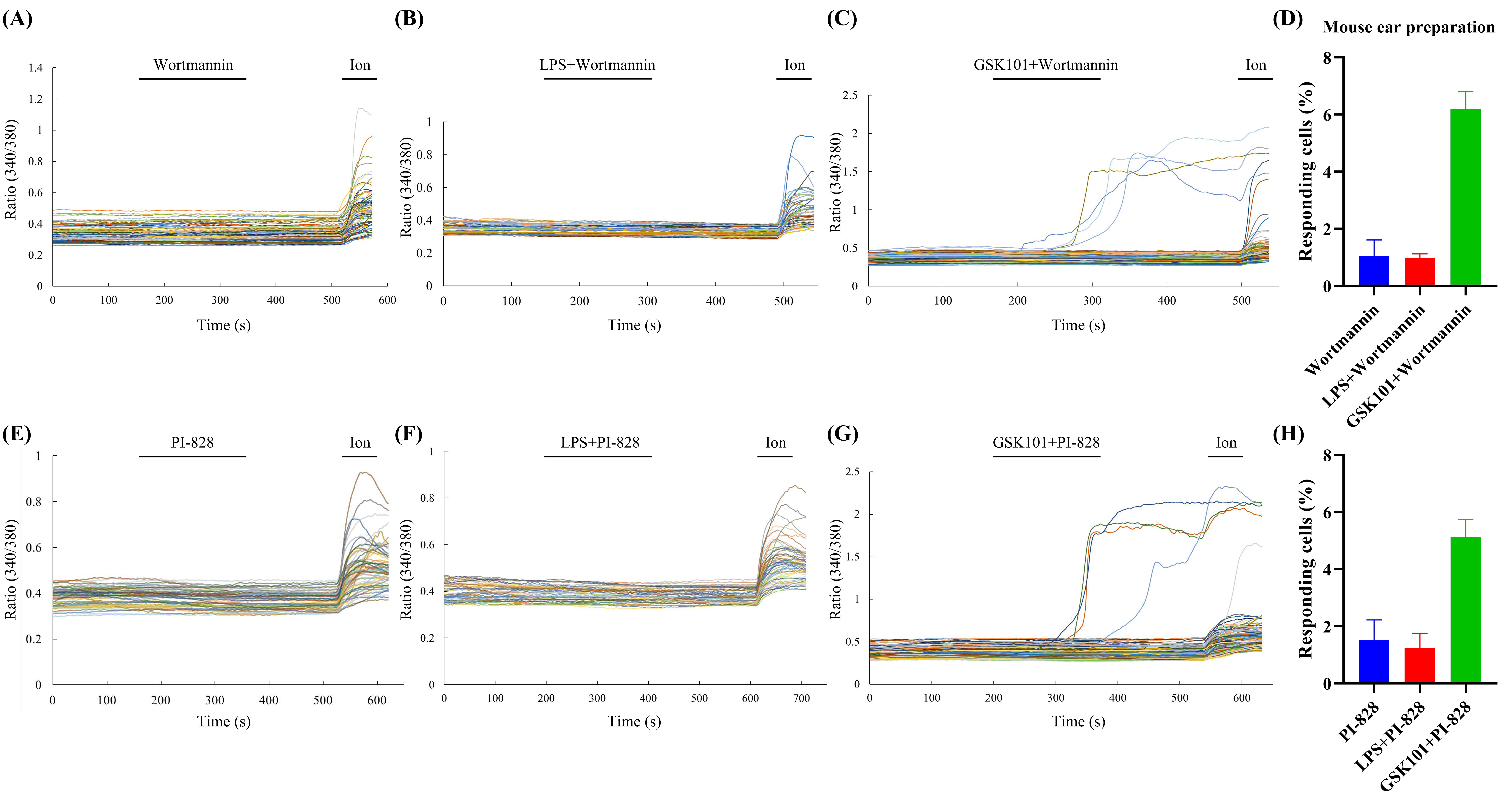

Supplement: Supplementary file 1 — Figures S1–S4. [file JCMM-28-e18509-s001.zip › jcmm18509-sup-0002-FigureS2.jpg]

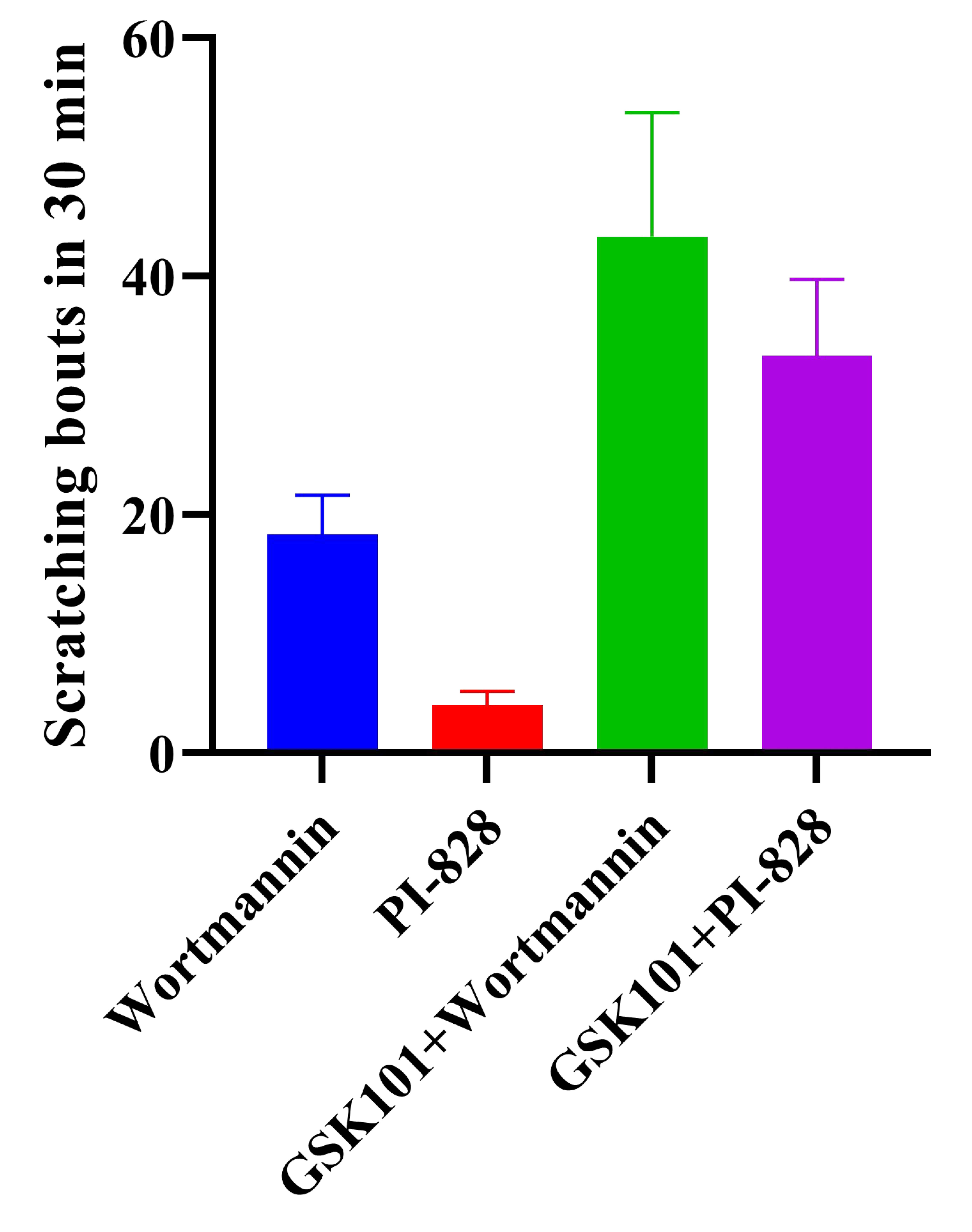

Supplement: Supplementary file 1 — Figures S1–S4. [file JCMM-28-e18509-s001.zip › jcmm18509-sup-0003-FigureS3.jpg]

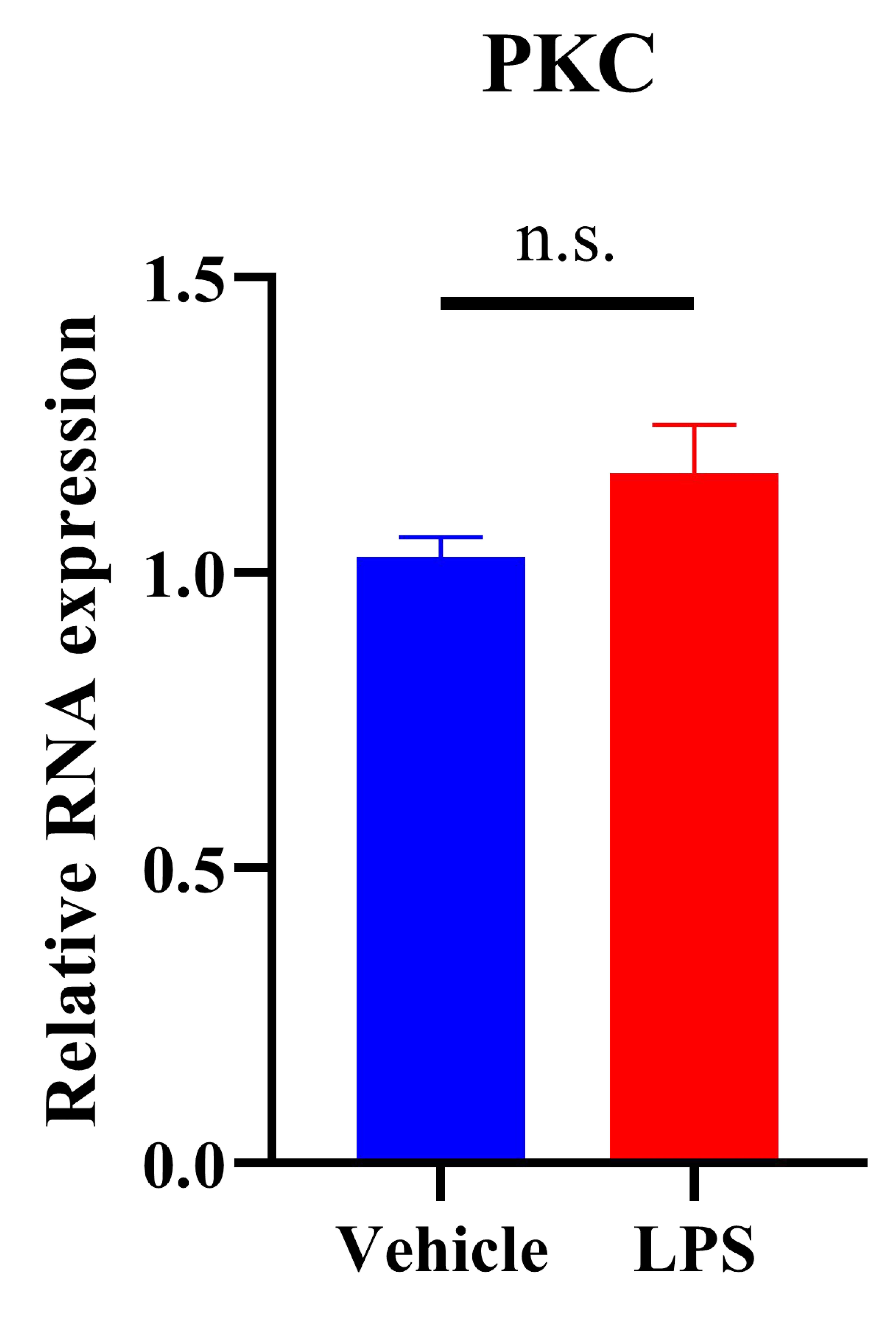

Supplement: Supplementary file 1 — Figures S1–S4. [file JCMM-28-e18509-s001.zip › jcmm18509-sup-0004-FigureS4.jpg]
